# MAGED2 Is Required under Hypoxia for cAMP Signaling by Inhibiting MDM2-Dependent Endocytosis of G-Alpha-S

**DOI:** 10.3390/cells11162546

**Published:** 2022-08-16

**Authors:** Elie Seaayfan, Sadiq Nasrah, Lea Quell, Maja Kleim, Stefanie Weber, Hemmo Meyer, Kamel Laghmani, Martin Kömhoff

**Affiliations:** 1 University Children’s Hospital, Philipps University, 35043 Marburg, Germany; 2 Faculty of Biology, University of Duisburg-Essen, 45141 Duisburg, Germany; 3 Centre de Recherche des Cordeliers, Sorbonne Université, Inserm, Université de Paris, CNRS, ERL8228, F-75006 Paris, France

**Keywords:** MAGED2, hypoxia, G-alpha-S, MDM2, Bartter

## Abstract

Mutations in *MAGED2* cause transient Bartter syndrome characterized by severe renal salt wasting in fetuses and infants, which leads to massive polyhydramnios causing preterm labor, extreme prematurity and perinatal death. Notably, this condition resolves spontaneously in parallel with developmental increase in renal oxygenation. MAGED2 interacts with G-alpha-S (Gαs). Given the role of Gαs in activating adenylyl cyclase at the plasma membrane and consequently generating cAMP to promote renal salt reabsorption via protein kinase A (PKA), we hypothesized that MAGED2 is required for this signaling pathway under hypoxic conditions such as in fetuses. Consistent with that, under both physical and chemical hypoxia, knockdown of MAGED2 in renal (HEK293) and cancer (HeLa) cell culture models caused internalization of Gαs, which was fully reversible upon reoxygenation. In contrast to Gαs, cell surface expression of the β2-adrenergic receptor, which is coupled to Gαs, was not affected by MAGED2 depletion, demonstrating specific regulation of Gαs by MAGED2. Importantly, the internalization of Gαs due to MAGED2 deficiency significantly reduced cAMP generation and PKA activity. Interestingly, the internalization of Gαs was blocked by preventing its endocytosis with dynasore. Given the role of E3 ubiquitin ligases, which can be regulated by MAGE-proteins, in regulating endocytosis, we assessed the potential role of MDM2-dependent ubiquitination in MAGED2 deficiency-induced internalization of Gαs under hypoxia. Remarkably, MDM2 depletion or its chemical inhibition fully abolished Gαs-endocytosis following MAGED2 knockdown. Moreover, endocytosis of Gαs was also blocked by mutation of ubiquitin acceptor sites in Gαs. Thus, we reveal that MAGED2 is essential for the cAMP/PKA pathway under hypoxia to specifically regulate Gαs endocytosis by blocking MDM2-dependent ubiquitination of Gαs. This may explain, at least in part, the transient nature of Bartter syndrome caused by *MAGED2* mutations and opens new avenues for therapy in these patients.

## 1. Introduction

Mutations in *MAGED2* associate with marked renal salt wasting in affected fetuses and newborns leading to extreme prematurity and increased perinatal mortality. Renal salt wasting is accompanied by massive polyuria and caused by defective salt reabsorption in the thick ascending limb of the loop of Henle and is referred to as transient Bartter syndrome (or Bartter V). Intriguingly, although patients with Bartter V are characterized by the most severe presentation due to profound excess fetal urine production causing preterm delivery, renal salt and water wasting resolves rapidly and completely starting at late gestation (30 weeks of gestational age) [1]. The reason for the spontaneous recovery, which occurs despite continuous expression of MAGED2 in the distal tubule from early fetal development into adulthood, is not known, suggesting that spontaneous recovery is triggered by an external factor or factors. Resolution of hypoxia may be the relevant external factor [1], because kidneys (both in utero as well as in extremely preterm babies) are subjected to hypoxia as evidenced by the expression of the hypoxia inducible factor (HIF-1α) until approximately the 30th week of gestation [2,3,4,5]. The renal medulla, where NKCC2, one of the key salt transport proteins (mutated in Bartter I) is expressed, has a low oxygen tension, which is further exacerbated during pregnancy in utero. Remarkably, this period coincides with the onset of recovery from salt and water wasting in transient Bartter syndrome. Furthermore, the notion that the function of MAGED2 is to protect renal salt transport against hypoxic stress is consistent with recent studies demonstrating that MAGE proteins protect against diverse forms of stress (radiation, genotoxic, and nutritional stress) [6] by regulating the activity of ubiquitin E3 ligases [7], of which ca. 650 members are known. E3 ligases are the ultimate enzymes involved in the transfer of ubiquitin to substrate proteins, a process that determines the fate of the modified protein. In this regard, it is worth noting that E3 ligases can promote not only the ubiquitination-dependent degradation of client proteins but also a non-degradative ubiquitination pathway involved in the regulation of proteins’ endocytosis and trafficking [8].

We and others have recently demonstrated that MAGED2 protein interacts with Gαs [1,9]. Gαs transmits activation of G-protein coupled receptors (GPCR) resulting in activation of membranous adenylate cyclase and hence cAMP formation [10]. In the kidney, cAMP generation is downstream of vasopressin and other hormones promoting renal tubular salt and water reabsorption [11]. Of note, NKCC2 (mutated in Bartter I) and NCC are two important renal salt transporters, whose intrarenal expression and function are greatly reduced in Bartter V [1] and are stimulated by cAMP [11].

In this study, we hypothesized that the interaction of MAGED2 and Gαs is essential under hypoxic conditions such as in fetal kidneys to allow for sufficient cAMP generation and hence activation of the PKA pathway to promote salt reabsorption. We therefore examined the effects of MAGED2 knockdown on the intracellular localization of Gαs, and the cAMP/PKA pathway with and without physical and chemical hypoxia in a renal cell culture system (HEK293) and in a human cancer cell line (HeLa). We found that, under hypoxia, MAGED2 prevents translocation of Gαs from the plasma membrane to the cytosol by blocking the E3 ligase MDM2, which triggers endocytosis of Gαs. Accordingly, MAGED2 depletion impaired cAMP generation and PKA activity. Thus, we demonstrated that MAGED2 is required for the activation of the cAMP/PKA pathway under hypoxic conditions to regulate Gαs endocytosis via MDM2-dependent ubiquitination, thereby explaining, at least in part, the transient nature of transient Bartter syndrome.

## 2. Materials and Methods

### 2.1. Plasmid Constructions and Site Directed Mutagenesis

The V5 tagged Gαs was generated by Site-Directed Mutagenesis using the long isoform as template according to the Q5^®^ Site-Directed Mutagenesis Kit protocol. To generate K28R, K53R, K88R, K300R, and K305R mutations, the site-directed mutagenesis method was performed using wild-type Long Gαs-HA construct as a template according to the QuikChange Multi Site-Directed Mutagenesis Kit protocol. All mutations were confirmed by sequencing.

### 2.2. Cell Culture

Human Embryonic Kidney (HEK293) and HeLa cells (Table 1) were grown in DMEM Glutmax complemented with 10% fetal bovine serum superior (Sigma-Aldrich, Schnelldorf, Germany), penicillin (100 units/mL), and streptomycin (100 units/mL) at 37 °C in a humidified atmosphere containing 5% CO_2_. For chemical treatment experiments, the media of confluent cells was changed to DMEM serum free for 14–16 h. Cells for control and experimental groups are always derived from the same flask and passage and studied on the same day.

### 2.3. Chemical Hypoxia

Chemical hypoxia was induced by cobalt chloride (CoCl_2_). For hypoxic incubation, media of confluent cells was changed to DMEM without serum supplemented by 300 µM CoCl_2_ or the specified dose for the dose response experiment. Cells were placed in a standard humidified at 37 °C for 14–16 h. Induction of hypoxia was confirmed by Western blotting for HIF-1α protein expression.

### 2.4. Physical Hypoxia

Physical hypoxia of cells was performed in a modular hypoxia incubator chamber (Billups-Rothenberg, Inc., San Diego, CA, USA, Cat. MIC-101). For hypoxic incubation, after cells became confluent, media was changed to DMEM without serum and cells were placed in the center of the chamber, which was sealed shut and connected via a single flow meter (Billups-Rothenberg, Inc., San Diego, CA, USA, Cat. SFM-3001) to a gas tank containing 1% O_2_, 5% CO_2_, and 94% N_2_. The modular chamber was placed in a standard humidified incubator at 37 °C for 14–16 h or the specified time for the time course experiment. For the recovery experiment, 100 µg/mL cycloheximide was added to the cells before being placed outside the hypoxia chamber for 2 h to prevent protein synthesis. A normoxic control was placed in the same incubator outside of the hypoxia chamber. Induction of hypoxia was confirmed by Western blotting for HIF-1α protein expression.

### 2.5. Small Interfering RNA (siRNA) Knockdown and Plasmid Transfection

The siRNAs for control, MAGE-D2, GNAS and MDM2 siRNAs were purchased from Dharmacon as ON-TARGETplus SMARTpools (D-001810-10-05 and L-010825-00-0005). Cells were first transfected with control or specific siRNA with Lipofectamine RNAiMAX (Invitrogen, Dreieich, Germany) by reverse transfection using the manufacturer’s specifications. Gαs-HA and Flag-β2AR expression plasmids were cotransfected with control or MAGE-D2 siRNAs with lipofectamine 3000 by reverse transfection using the manufacturer’s specifications.

### 2.6. Biotinylation

After hypoxia, confluent cells were washed twice with DPBS supplemented by 1 mM MgCl_2_ and 0.1 mM CaCl2 (PBS^++^). Cells were incubated at 4 °C for 30 min in PBS^++^ containing 1 mg/mL EZ-Link™ Sulfo-NHS-LC-Biotin. Cells were rinsed three times in PBS^++^ with 100 mM glycine and reincubated at 4 °C in the same solution for 10 min. Then, they were washed three times with PBS^++^. Washed cells were lysed for 45 min at 4 °C in solubilizing buffer (150 mm NaCl, 5 mm EDTA, 3 mm KCl, 120 mm Tris/Hepes, pH 7.4, 1% (*v*/*v*) Triton X-100) containing protease inhibitors (Sigma, Schnelldorf, Germany). After taking an aliquot of the total cell extract from each sample to provide a measure of total expression, the rest of cell lysates were incubated with neutravidin beads (Thermo Scientific™, 63303 Dreieich, Germany) overnight at 4 °C. After overnight incubation, samples were centrifuged at 13,000 rpm for 5 min, and the supernatant (the intracellular fraction) was removed. Neutravidin beads were then washed with solubilizing buffer and then centrifuged for 5 min at 13,000 rpm seven times. Pellets were incubated in Western blot loading buffer for 10 min at 95 °C and stored at −20 °C. Each fraction was subjected to SDS-PAGE and Western blot analysis.

### 2.7. Immunocytochemistry

Cells were fixed after biotinylation with 4% paraformaldehyde in PBS for 20 min at 4 °C, permeabilized with 0.1% Triton X-100 for 5 min at 4 °C and incubated with DAKO (antibody diluent with background-reducing components) for 30 min to block nonspecific antibody binding. Fixed cells were incubated for 1 h at room temperature with the primary antibody mouse anti-HA (1:50) in DAKO. Mouse anti-HA and biotinylated membrane proteins were visualized with Alexa 555-coupled secondary antibody (1:1000), and Alexa 488-coupled neutravidin (1:500), respectively. Cells were then washed three times with PBS and mounted with Vectashield containing Dapi. Cells were visualized using a Leica confocal (SP8i) microscope.

### 2.8. Immunoprecipitation Assay

Cells were lysed in TNTE buffer (20 mM Tris–HCl, pH 7.4, 150 mM NaCl, 5 mM EDTA, 0.5% Triton X-100, and 10% glycerol) in the presence of proteinase inhibitor cocktail (Sigma, Schnelldorf, Germany). Cell lysates’ (500 μg total) immunoprecipitation was performed with the primary antibody of interest coupled and crosslinked, using the crosslinker BS^3^ (bis(sulfosuccinimidyl)suberate), to the protein G magnetic beads (Dynabeads). After incubation with magnetic protein G beads coupled to the indicated antibody for 1 h at room temperature, the immune complex was washed three times in PBS (Invitrogen, Dreieich, Germany). The protein samples were boiled in loading buffer, separated on 7.5% TGX Stain-Free gel, and probed with the primary antibodies of interest and fluorescence conjugated secondary antibodies according to standard procedures.

### 2.9. Ubiquitination Assay

Cells were transfected by control or MAGE-D2 siRNA by reverse transfection using lipofectamine RNAiMAX. The second day, cells were transfected with WT V5 tagged Gαs with single ubiquitin tagged HA. Later, cells were exposed overnight to hypoxia and lysed using RIPA buffer (50 mM Tris/HCl, 150 mM NaCl, 1 mM EDTA, 0.1% SDS, 1% Triton X-100, and 100 mM N-ethylmaleimide). Cell lysates were cleared at 13,000× *g* for 15 min. Protein concentrations of the supernatants were determined using a Pierce™ BCA Protein Assay Kit (Thermo Scientific™, Dreieich, Germany). The samples were then subjected to immunoprecipitation using protein G magnetic beads coupled to anti-V5 antibody, as described in the co-immunoprecipitation section.

### 2.10. Intracellular cAMP Measurement

Cyclic AMP was measured using a cAMP complete in vitro ELISA kit (Abcam, Berlin, Germany, ab133051), in which a goat anti-rabbit immunoglobulin G binds with a cAMP antibody. Cyclic AMP then binds to the antibody in competition with a labelled colorimetric conjugate, which was measured at 405 nm using a microplate reader (Tecan Infinite Pro). Standards of known cAMP concentrations were used to compare to samples. Before the assay, cell lysates samples prepared in 0.1 M HCL containing 0.1% Triton X-100. The homogenate was pelleted, and the supernatant was used for the assay. Two blanks were included, one with substrate only, and the other received all additions except a sample. Control wells were also monitored for non-specific binding and colorimetric maxima.

### 2.11. PKA Kinase Activity

The PepTag Nonradioactive Protein Kinase Assay Kit (Promega, Madison, WI, USA) was used according to the manufacturer’s instructions. Quantification of the phosphorylated peptide substrate was performed by spectrophotometry by comparing the absorbance at 570 nm.

### 2.12. Western Blotting

After three washes with ice-cold phosphate-buffered saline (PBS), cells were lysed in lysis buffer (50 mM Tris pH 7.4, 5 mM EDTA, 150 mM NaCl, 1% Triton X-100, and protease inhibitors) and cell lysates were cleared at 13,000× *g* for 15 min. Protein concentrations of the supernatants were determined using a Pierce™ BCA Protein Assay Kit (Thermo Scientific™, Dreieich, Germany). Proteins were separated in 7.5% TGX Stain Free gels (Bio-rad, Feldkirchen, Germany, Cat. 1610181) and after transferring to nitrocellulose membrane (Bio-rad, Feldkirchen, Germany, Cat. 1704270) using a Trans-Blot Turbo Transfer System (Bio-rad, Feldkirchen, Germany), proteins were detected with fluorescently labeled antibodies StarBright Blue 520 and 700 (Bio-rad, Feldkirchen, Germany). Imaging of the blots were performed using a ChemiDoc MP (Bio-Rad, Feldkirchen, Germany). Gray density of Western blots was measured using ImageJ software (National Institutes of Health, Bethesda, MD, USA).

### 2.13. Statistical Analyses

Results are expressed as mean ± SEM Differences between means were evaluated using unpaired Student t test. Statistical analyses were performed using GraphPad Prism X9 software. *p* ≤ 0.05 was considered statistically significant (*), *p* ≤ 0.01 was considered highly significant (**) and *p* ≤ 0.001 was considered very highly significant (***).

## 3. Results

### 3.1. MAGED2 Is Required for the Expression of Gαs at the Plasma Membrane under Hypoxic Condition

**Figure 1:**
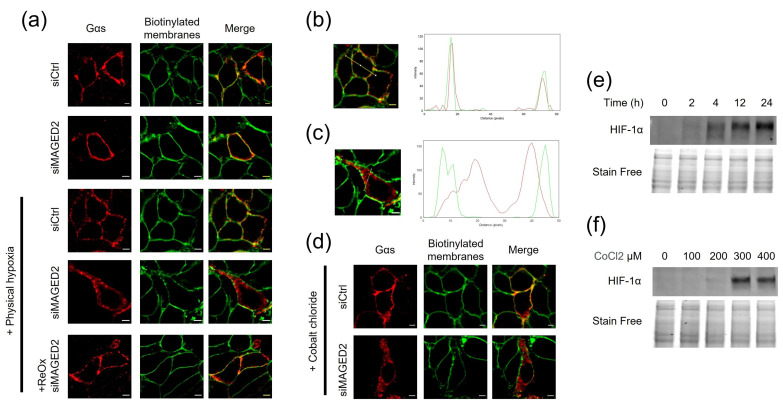
MAGED2 prevents internalization of Gαs under hypoxic condition. (**a**) Immunolocalization studies of Gαs proteins in presence and absence of MAGED2. HeLa cells were co-transfected with a Gαs-HA construct and control or MAGED2 siRNA. Forty-eight hours post-transfection, growth medium was replaced by DMEM serum free and cells were exposed to physical hypoxia (1% oxygen overnight), as indicated. Membrane proteins of HeLa cells were biotinylated at 4 °C. Scale bars, 5 μm. (**b**,**c**) Using the “RGB profile plot” plugin in ImageJ, we determined the pattern distribution of Gαs (red) in comparison to the biotinylated membrane proteins (Green) in the absence (**b**) or presence (**c**) of MAGED2 siRNA under hypoxia. (**d**) Immunolocalization studies of Gαs proteins in presence and absence of MAGED2 under chemical hypoxia. HeLa cells were co-transfected with a Gαs-HA construct and control or MAGED2 siRNA. Forty-eight hours post-transfection, growth medium was replaced by DMEM serum free and exposed to chemical hypoxia (300 µM CoCl_2_), as indicated. Membrane proteins of HeLa cells were biotinylated at 4 °C. Scale bars, 5 μm. (**e**) cells were treated with physical hypoxia (1% O_2_, 5% CO_2_, 94% N_2_) for the specified times. (**f**) cells were treated with chemical hypoxia with the indicated dose of CoCl_2_ for 14–16 h. Chemical hypoxia. (**e**,**f**) Total cell lysates were separated by SDS-PAGE and probed with anti-HIF-1α antibodies.

We first asked whether MAGED2 regulated the localization and hence function of Gαs. To address this question, we monitored intracellular distribution of transiently expressed Gαs in control- or MAGED2-depleted HeLa cells by immunocytochemistry. The plasma membrane was labelled with a biotinylated fluorophore. As illustrated in Figure 1a, MAGED2 knockdown did not affect the expression of Gαs at the cell surface under normoxia. In contrast, in cells exposed to physical hypoxia (1% oxygen overnight), MAGED2 knockdown resulted in a dramatic change of the subcellular localization of Gαs from the plasma membrane to an intracellular localization (Figure 1a–c and Figure S1), with both a diffuse and a vesicular pattern. Interestingly, similar results were obtained with overexpressed (Figure 1d and Figure S2) and endogenous (Figure S3) Gαs, in HEK293 and HeLa cells, when cellular hypoxia was induced chemically for 16 h with 300 µM of cobalt chloride (CoCl_2_) which acts as a hypoxia mimetic by inhibiting prolyl hydroxylase activity [15]. Notably, Gαs membrane expression was restored when cells were exposed to normoxia for 2 h. It is worth noting that this restoration occurred in the presence of cycloheximide, a protein synthesis inhibitor, which was added to exclude the potential implication of newly synthesized proteins. Of note, the marked induction of HIF-1α protein levels by Western blotting in response to physical and chemical hypoxia demonstrates that relative hypoxia was achieved (Figure 1e,f).

### 3.2. Expression of the β2-Adrenergic Receptor at the Plasma Membrane Is Independent of MAGED2

**Figure 2:**
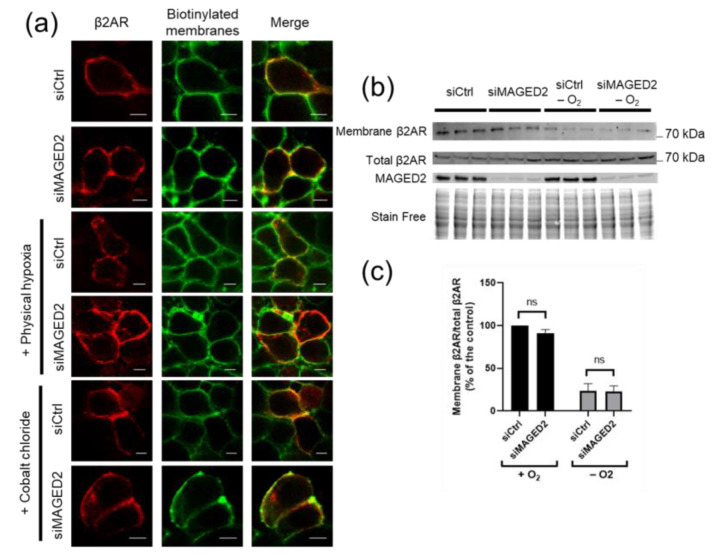
β2-adrenergic receptor internalization is independent of MAGED2 under hypoxic condition. (**a**) Immunolocalization studies of β2-adrenergic receptor (β2AR) in the presence and absence of MAGED2. HeLa cells were co-transfected with a Flag-β2AR construct and control or MAGED2 siRNA. Forty-eight hours post-transfection, growth medium was replaced by DMEM serum free and exposed to physical hypoxia (1% oxygen overnight) or chemical hypoxia (300 µM CoCl_2_), as indicated. Membrane proteins of HeLa cells were biotinylated at 4 °C. Scale bars, 5 μm. (**b**) HeLa cells were transfected with control and MAGED2 siRNA. In 24–48 h post-transfection, cells were treated with physical hypoxia. Cell surface biotinylated proteins were recovered from cell extracts by precipitation with neutravidin-agarose. β2AR on the cell surface were detected by Western blotting with a β2AR antibody. An aliquot of the total cell extract from each sample was also run on a parallel SDS gel and Western blotted for total β2AR and MAGED2 expression. (**c**) Densitometric analysis of (**b**), shown as ratio of membrane β2AR and total β2AR immunoblot. Bar graphs show mean ± SEM.

We next investigated whether the MAGED2 deficiency-induced down regulation of Gαs cell surface expression under hypoxia can also affect the β2-adrenergic receptor, which is known to interact physically and functionally with Gαs. To this end, we assessed endogenous (Figure 2b,c) and transiently (Figure 2a) expressed β2-adrenergic receptor surface expression using immunocytochemistry and cell surface biotinylation assays. As can be observed in Figure 2a, immunocytochemistry staining shows that MAGED2 knockdown does not affect the cell surface expression of the β2-adrenergic receptor under normoxic or hypoxic (physical and chemical hypoxia) conditions as judged by the colocalization of the β2-adrenergic receptor with biotinylated plasma membrane proteins. As expected, immunoblot analysis shows that, similar to the immunocytochemistry experiments, cell-surface expression of endogenous β2-adrenergic receptor was not reduced upon MAGED2 knockdown under hypoxia (Figure 2b,c), clearly demonstrating that the effect of MAGED2 knock-down on Gαs localization under hypoxia is specific. Importantly, we observed internalization the β2-adrenergic receptor under hypoxic conditions-independently of MAGED2 knockdown, which can be explained by activation of the receptor under hypoxia [16,17].

### 3.3. MAGED2 Is Required for cAMP Generation and PKA Activity under Hypoxia

**Figure 3:**
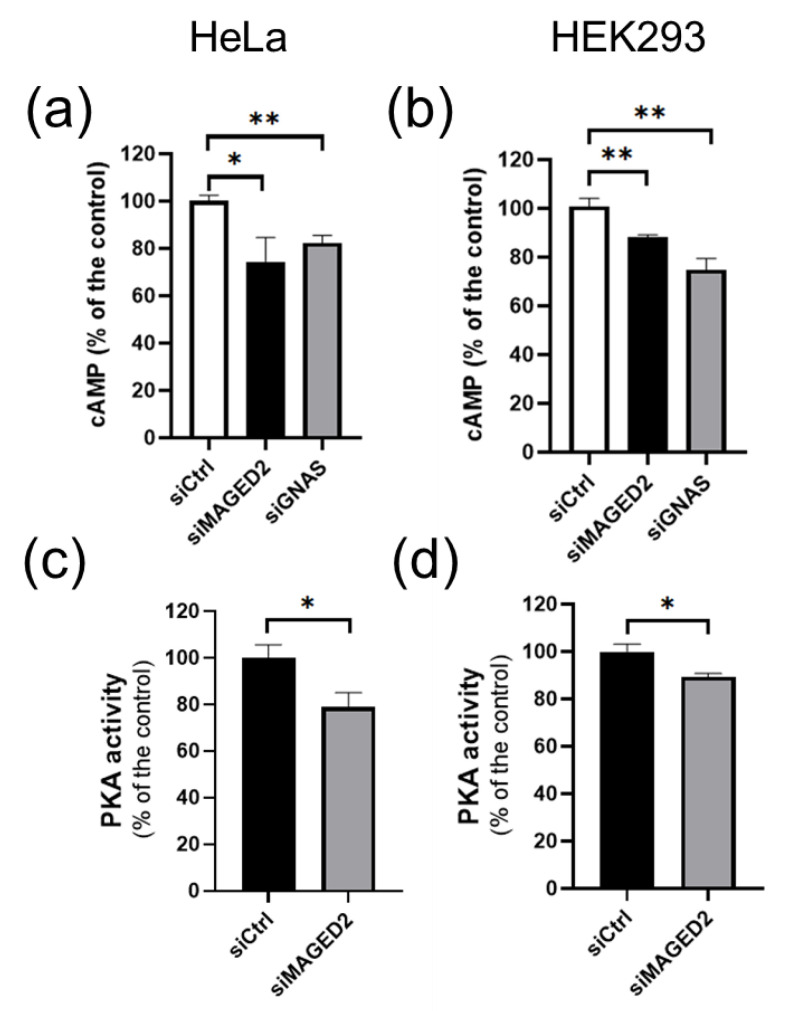
MAGED2 depletion decreases cAMP production and PKA activity under hypoxic condition. HeLa (**a**,**c**) and HEK293 (**b**,**d**) cells were transfected with control, MAGED2 and Gαs siRNA. In 24–48 h post-transfection, growth medium was replaced by DMEM serum free supplemented with 300 µM Cobalt chloride (CoCl_2_). (**a**,**b**) Cells were lysed with 0.1 M HCL containing 0.1% Triton X-100 and cAMP was measured by ELISA. (**c**,**d**) Cells were lysed with PKA extraction buffer and PKA activity was measured with the PepTag Nonradioactive Protein Kinase Assay Kit. Statistical significance was determined by unpaired two-sided Student’s t tests. All data are shown as a representative result from three independent experiments. Bar graphs show mean ± SEM. * *p* ≤ 0.05 and ** *p* ≤ 0.01.

Gαs localizes to the plasma membrane to signal GPCR activation to membranous adenylate cyclase in order to promote generation of cAMP and augment PKA activity. Hence both processes could be affected by MAGED2 depletion. We therefore assessed intracellular cAMP levels by ELISA in the different conditions. HeLa and HEK293 cells were transfected by control or MAGED2 siRNA, and subsequently subjected to a hypoxic microenvironment chemically induced by 300 µM of CoCl_2_ or physically induced by exposure to 1% oxygen for 16 h. As shown in Figure 3a,b and Figure S4a,c, MAGED2 knockdown decreased basal cAMP levels in HeLa and HEK293 cells, respectively. This decrease was unaffected by the addition of IBMX (Figure S4b), a phosphodiesterase inhibitor, but reversed by the addition of forskolin, showing that MAGED2 regulates the cAMP level by regulating the localization of Gαs without affecting the activity of adenylate cyclase or phosphodiesterase. Under the same conditions, MAGED2 knockdown reduced PKA activity (Figure 3c,d). In a parallel set of experiments, we analyzed the effect of Gαs knockdown. Gαs knockdown mirrored the effect of MAGED2 depletion, as it reduced basal cAMP levels in both HeLa and HEK293 cells (Figure 3a,b and Figure S4a,b).

### 3.4. Under Hypoxia, MAGED2 Depletion Promotes Endocytosis of Gαs by Enhancing Its MDM2-Dependent Ubiquitination

**Figure 4:**
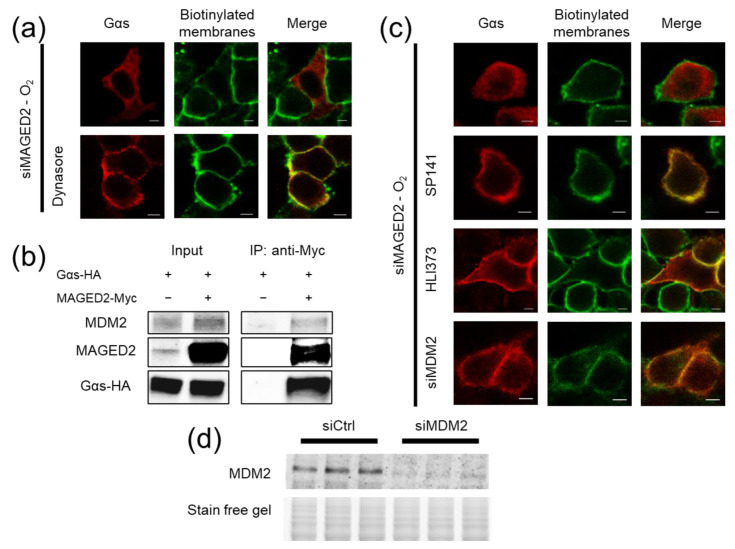
MAGED2 prevents MDM2-mediated internalization of Gas under hypoxia. (**a**) HeLa cells were co-transfected with Gαs-HA construct and MAGED2 siRNA. Forty-eight hours post-transfection, cells were treated with physical hypoxia overnight in the presence or absence of an endocytosis inhibitor, Dynasore 50 µM, as indicated. The stained specimens were evaluated by ApoTome microscopy. Scale bars, 5 μm. (**b**) HeLa cells transiently transfected with a Gαs-HA construct alone or in combination with a MAGED2-Myc construct were immunoprecipitated (IP) with anti-Myc antibodies. A total of 5% of total cell lysate was loaded for comparison. Co-immunoprecipitated Gαs, MAGED2, and MDM2 proteins were detected by immunoblotting using anti-MAGED2, anti-HA, and anti-MDM2 antibodies, respectively. (**c**) HeLa cells were co-transfected with Gαs-HA construct and MAGED2 siRNA with or without MDM2 siRNA. Forty-eight hours post-transfection, cells were treated with physical hypoxia overnight in the presence or absence of MDM2 inhibitors, SP141 (1 µM), or HLI373 (3 µM) as indicated. The stained specimens were evaluated by ApoTome microscopy. Scale bars, 5 μm. (**d**) Cells were transfected with control or MDM2 siRNA as indicated. In 24 h post-transfection, cells were lysed in RIPA buffer and cell lysates were separated by SDS-PAGE and probed by anti-MDM2 antibody.

Because Gαs subcellular redistribution under hypoxia suggests that MAGED2 knock-down enhances Gαs internalization, we first studied the effect of dynasore, a small molecule inhibitor of dynamin mediated endocytosis. As shown in Figure 4a, dynasore treatment fully rescued membrane expression of Gαs upon of MAGED2 depletion. Altogether, the above data provide evidence that the decrease in Gαs cell surface expression was due to an enhanced internalization of the protein, pointing therefore to an inhibitory effect of MAGED2 on endocytosis of Gαs under hypoxia.

The endocytosis of many proteins is regulated by the action of E3 ubiquitin ligases in a non-degradative fashion by a process called mono or multi-mono ubiquitination [8,18,19]. MAGED2 has been described to interact with both the E3 ubiquitin ligase MDM2 [1,20,21,22] and Gαs [23]. As MDM2 has also been shown to regulate Gαs [23], we hypothesized that MAGED2 regulates Gαs endocytosis via MDM2. Confirming this notion, we first recapitulated the interaction of MAGED2 to both Gαs and MDM2 by Co-IP experiments (Figure 4b). Their interaction is in keeping with the notion that Gαs is ubiquitinated by MDM2 in a MAGED2 dependent fashion.

**Figure 5:**
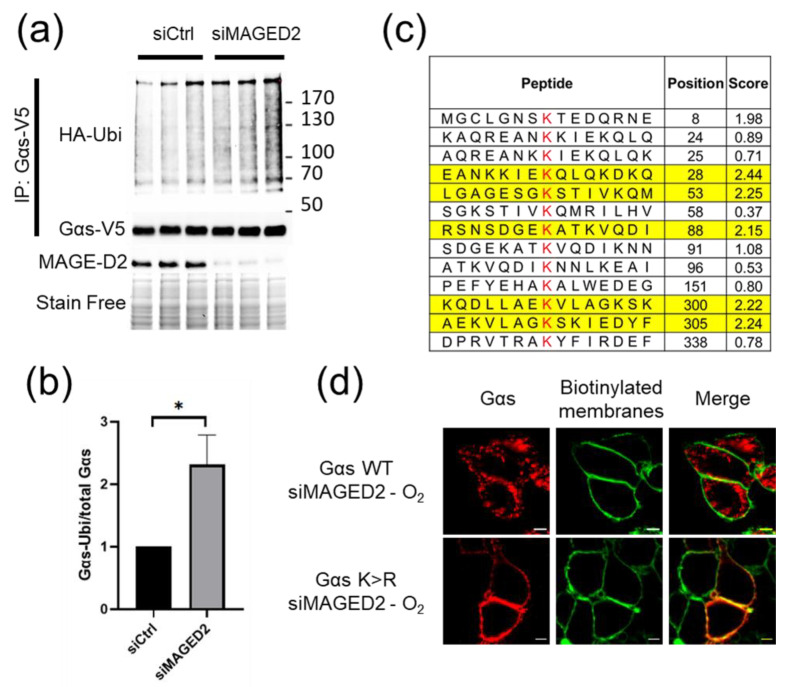
MAGED2 inhibits Gαs ubiquitination under hypoxic condition. (**a**) MAGED2 regulates Gαs ubiquitination. HEK293 cells, transiently transfected with Gαs-V5 and ubiquitin-HA, were immunoprecipitated with anti-V5 under denaturing conditions. Ubiquitinated Gαs was detected with anti-HA antibody. (**b**) Densitometric analysis of (**a**), shown as ratio of ubiquitinated Gαs and total Gαs immunoblot. Statistical significance was determined by unpaired two-sided Student’s *t*-tests. Data are shown as a representative result from three independent experiments. Bar graphs show mean ± SEM. * *p* ≤ 0.05. (**c**) The ubiquitination sites of Gαs were predicted with a Bayesian Discriminant Method [24] and the predicted sites with a score ≥ 2 were chosen for mutation (yellow). (**d**) Immunofluorescence microscopy showing distribution of wild type (WT) or a Gas-HA variant harboring 5 lysine-to-arginine substitutions (5×K > R) in HeLa cells under physical hypoxia. Cells were stained with mouse anti-HA antibody for Gαs (Alexa 555, Red) and plasma membrane biotinylated proteins (Alexa 488, Green). The yellow color (merged image) indicates co-localization of the proteins. Scale bars, 5 μm.

To gain experimental evidence that Gαs is ubiquitinated in a MAGED2-dependent fashion, we combined expression of HA-tagged ubiquitin and V5-tagged Gαs with knockdown of MAGED2 followed by physical hypoxia (Figure 5a, lower part). Immunoblot analysis of Gαs immunoprecipitates using an HA-antibody, revealed increased staining intensity upon knockdown of MAGED2 (Figure 5a, upper part). Given the comparable amounts of immunoprecipitated total V5-Gαs proteins (Figure 5a, middle part), our data strongly indicate that ubiquitination of Gαs under hypoxia is markedly increased upon knockdown of MAGED2, which could be confirmed by densitometric analysis (Figure 5b).

After having shown that Gαs is ubiquitinated and endocytosed in a MAGED2 dependent fashion, we aimed to gain functional evidence that these processes are indeed regulated by MDM2. Therefore, we inhibited MDM2 by various approaches. As illustrated in Figure 4c, chemical inhibition of MDM2 with either Sp-141 or HLI-373 [25] rescued Gαs plasma membrane localization following MAGED2 depletion under hypoxia. Importantly, MDM2 knockdown reproduced the same effect (Figure 4c). Of note, a marked reduction of MDM2 protein levels with siRNA was confirmed by Western blotting (Figure 4d). Consistent with abrogating internalization of Gαs in MAGED2-depleted hypoxic cells, Sp-141 treatment also prevented the decrease in cAMP caused by MAGED2 knockdown under physical hypoxia (Figure S4b).

Finally, to prove that ubiquitination of Gαs is necessary to allow its endocytosis, we chose five (out of thirteen) lysine residues of Gαs protein that could serve as acceptor sites for ubiquitin based on their accessibility and mutated them to arginine (5×K > R) (Figure 5c). Immunocytochemistry of cells expressing the 5×K > R Gαs variant demonstrated that endocytosis triggered by hypoxia and MAGED2 knockdown was suppressed by the mutations compared to wild type Gαs (Figure 5d). To exclude the possibility that the 5×K > R Gαs construct is locked in the plasma membrane because of a general defect of internalization, cells were treated with cholera toxin, which induces Gαs internalization by directly activating its GTPase function [26]. As shown in Figure S6, the cholera toxin induced internalization of both wildtype and the mutant 5×K > R Gαs variant. This finding supports the notion that the blockade of the hypoxia-induced endocytosis of Gαs in MAGED2-depleted cells results specifically from impaired ubiquitination of critical lysine residues and not from a general defect in protein internalization (Figure S6). Taken together, our data clearly demonstrate that MAGED2 deficiency leads to the ubiquitin and MDM2-triggered endocytosis of Gαs under hypoxic conditions.

## 4. Discussion

**Figure 6:**
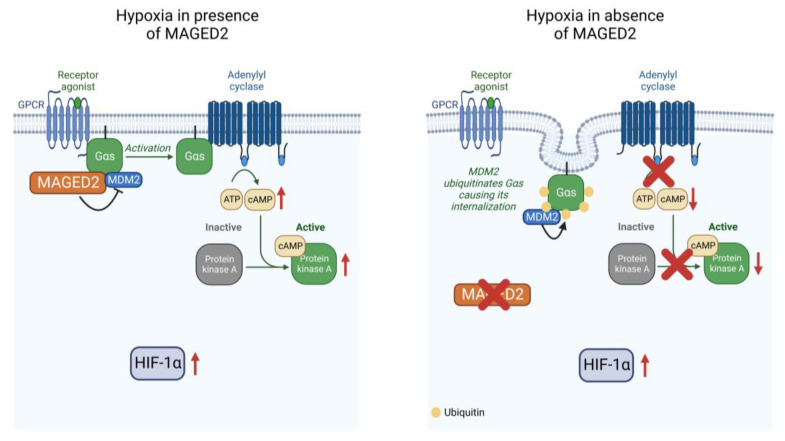
Proposed model for MAGED2′s role under hypoxia (created with BioRender.com). Under hypoxia, MAGED2 inhibits MDM2 dependent ubiquitination and endocytosis of Gαs. This ensures activation of the adenylate cyclase and cAMP generation and activation of PKA under hypoxia. Reduced cAMP levels impair cAMP-dependent salt reabsorption, explaining salt wasting in transient Bartter syndrome.

The molecular basis of the role of MAGED2 in transient Bartter syndrome, which is characterized by profound fetal salt wasting and polyuria leading to perinatal death and extreme prematurity followed by spontaneous recovery in the survivors, has been unknown. In this study, we demonstrate that MAGED2 acts as a master regulator of cAMP/PKA under hypoxia, by controlling the endocytosis Gαs via MDM2-dependent ubiquitination (Figure 6). Because the essential salt-transporters NKCC2 and NCC require cAMP for proper expression and functioning our finding of Gαs mistargeting and impaired cAMP generation in MAGED2-depleted hypoxic cells may explain, at least in part, the transient nature of antenatal Bartter syndrome caused by MAGED2 mutations and reveal potential strategies of intervention in this disease and beyond.

On the cell biological level, we now demonstrate that MAGED2 is necessary under hypoxia to prevent ubiquitin-dependent and dynasore-sensitive endocytosis of Gαs by blocking the E3 ubiquitin ligase MDM2. The mechanism how Gαs dissociates from the plasma membrane to enter into the endocytic network, where it engages with GPCRs and the epidermal growth factor receptor (EGFR) to promote signaling [27,28] and sorting [29,30], respectively, is ill defined. The depalmitoylation of Gαs followed by simple diffusion through the cytosol was proposed [31]. Indeed, imaging studies have demonstrated that internalized Gαs can appear to be diffuse in the cytosol [26,32], but association with intracellular vesicles has also been observed [33,34,35].

Given the well-recognized role of ubiquitination in endocytosis [8] our finding that Gαs is internalized in an ubiquitin dependent manner is—although not unexpected—a necessary step to pave the way for subsequent studies, giving more insight into the regulation of the endocytosis of Gαs. We demonstrate that MDM2 is the relevant E3 ligase mediating the MAGED2 dependent endocytosis of Gαs, which concurs with previous studies demonstrating a physical interaction between MDM2 and MAGED2 [1,22], as well as MAGED2 and Gαs [1,9]. Inhibition of ubiquitination and endocytosis of Gαs could result from MAGED2 inhibiting the ligase activity of MDM2, a mechanism also proposed for MAGEA2 and MAGEC2 [36,37]. Alternatively, MAGED2 could inhibit ubiquitination of Gαs by shielding it from the ligase activity of MDM2. Our data thus corroborate and extend previous studies on the important role of MDM2 in the regulation of GPCR-signaling, which demonstrated that the ubiquitination of β2-arrestin (ARRB2) by MDM2 promotes the endocytosis of the β2-adrenergic receptor under normoxia [38]. This demonstrates that MDM2 regulates GPCR signaling in a context and substrate specific way, which can be explained by MAGED2 acting as a specific adaptor of Gαs but not for the β2-adrenergic receptor, as the latter was internalized under hypoxia independently of the presence of MAGED2 (Figure 2). Targeting the cAMP/PKA cascade downstream of Gαs should be an ideal treatment for patients with transient Bartter syndrome: It could reactivate renal salt reabsorption to prevent perinatal death or sequalae such as intracerebral hemorrhage resulting from excessive amniotic fluid production causing preterm delivery. Although maternal hyperoxia results in increased oxygenation in the human fetus [39], which could stimulate the cAMP/PKA pathway and hence promote salt reabsorption, the toxicity of oxygen, especially in preterms resulting from the generation of reactive oxygen species, precludes its clinical use. Therefore, direct activators of the cAMP/PKA cascade such as forskolin, which can be given orally and has already been used in human clinical studies [40,41,42,43,44], could be studied in models of transient Bartter syndrome.

Our finding that MAGED2 can block MDM2-dependent internalization of Gαs may indicate that this mechanism is regulated in a graded fashion in normal subjects, perhaps by posttranslational modifications of MAGED2 to fine-tune MDM2 ligase activity in order to adjust the diverse functions of Gαs (and other substrates of MDM2).

By showing that MAGED2 is dispensable under normoxia (both in vivo and in vitro) but critically important under hypoxia to ensure the Gαs-dependent activation of cAMP/PKA pathway, our study adds hypoxia to the growing list of stressors (nutritional, genotoxic, and radiation stress) against which various members of the MAGE family have been shown to protect [6,45]. Of note, especially the renal medulla, where NKCC2, one of the key salt transport proteins, is expressed, has a low oxygen tension. The latter is even more severe in utero [2,3,4]. However, similar to the previously identified stressors, the molecular switch leading to inhibition of MDM2-dependent ubiquitination under hypoxia is unknown. Rapid relocation of Gαs to the plasma membrane upon reoxygenation in the presence of cycloheximide (which blocks protein synthesis) argues that the molecular switch under normoxia is brought about by posttranslational modification (s) of MDM2 and/or its partners.

Of note, MAGED2 is also expressed in many human cancers and is associated with a poor prognosis [4,46,47,48,49]. Moreover, the hypoxic microenvironment in cancer cells is the key condition affecting the cellular expression program, leading to chemotherapy resistance [50]. Given the established roles of Gαs as oncoprotein in malignancy [51,52,53], it is conceivable that MAGED2 promotes tumorigenesis by stimulating the cAMP/PKA pathway and could therefore be specifically targeted under hypoxia to inhibit the cAMP/PKA signaling.

The vast majority of patients with transient Bartter syndrome described so far have mutations corresponding to a (functional) knockout of the gene (19 out of 26 patients, please see table one in: [54]. These mutations include two deletions of the entire MAGED2 gene, and 17 mutations in the MAGED2 gene leading to premature termination codons >> 60 basepairs upstream of the 3’ most splice-generated exon-exon junction, which elicits nonsense-mediated decay (NMD). NMD is an essential RNA quality control mechanism that assures the quality of the transcriptome by eliminating transcripts that contain premature termination codons (PTCs) [55]. The remaining seven mutations include four missense mutations and three in frame deletions, which in general reduce the stability of the protein. We therefore think that the depletion of MAGED2 with our siRNA approach is a suitable model to study the function of MAGED2 at the cellular level for the majority of these patients.

As described above, we used HEK293 and HeLa cells instead of tubular renal cells to analyze the function of MAGED2 on Gαs signaling. Given that experiments yielded similar results in both cell lines, we are convinced that our findings can be generalized to human cells. We hypothesize that there is a specific phenotype of MAGED2 deficiency only in the kidney, because the renal medulla of the kidney is known for its low oxygen tension, which in combination with the physiological fetal hypoxia unveil its dependence of MAGED2 for proper Gαs dependent signaling under hypoxia.

In summary, we reveal that MAGED2 regulates ubiquitin dependent endocytosis of Gαs under hypoxia by inhibiting MDM2 and thereby acts a master regulator of the Gαs-dependent activation of the PKA pathway. Whereas activation downstream of Gαs could restore cAMP dependent salt reabsorption in transient Bartter syndrome, inhibition of MAGED2 could target the oncoprotein Gαs specifically in hypoxic tumors without disturbing Gαs signaling in normal tissue.

## Figures and Tables

**Table 1:** Reagent and tools.

Reagent or Resource	Source	Identifier
**Antibodies**		
Anti-HIF-1α rabbit	Cell Signaling	14179
Anti-MAGED2 rabbit	This paper	
Anti-beta 2 Adrenergic Receptor antibody	Abcam	ab182136
Anti-Gαs	Sigma Aldrich	06-237
Anti-HA tag mouse	Thermo Fisher Scientific	26183
V5-Tag antibody	Bio-rad	MCA1360GA
Monoclonal ANTI-FLAG^®^ M2 antibody produced in mouse	Sigma-Aldrich	F3165
Goat anti-Mouse IgG (H + L), Alexa Fluor Plus 555	Thermo Fisher Scientific	A32727
Streptavidin, Alexa Fluor™ 488 conjugate	Thermo Fisher Scientific	S11223
StarBright Blue 520 Goat Anti-Rabbit IgG	Bio-rad	12005869
StarBright Blue 700 Goat Anti-Mouse IgG	Bio-rad	12004158
**Chemicals, Peptides, and Recombinant Proteins**		
EZ-Link™ Sulfo-NHS-LC-Biotin	Thermo Fisher Scientific	21335
**Critical Commercial Assays**		
PepTag^®^ Non-Radioactive Protein Kinase Assays	Promega	V5340
cAMP Assay Kit (Competitive ELISA)	abcam	Ab133051
Q5^®^ Site-Directed Mutagenesis Kit	New England Biolabs’	E0554S
QuikChange Multi Site-Directed Mutagenesis Kit	Agilent Technologies	200515
**Experimental Models: Cell Lines**		
HEK293	ATCC	CRL1573
HeLa	Gift from Dr. Vijay Renigunta	
**Oligonucleotides**		
ON-TARGETplus Non-targeting Control Pool	Dharmacon	D-001810-10-05
UGGUUUACAUGUCGACUAA		
UGGUUUACAUGUUGUGUGA		
UGGUUUACAUGUUUUCUGA		
UGGUUUACAUGUUUUCCUA		
ON-TARGETplus Human MAGED2 siRNA—SMARTpool	Dharmacon	L-017284-01-0005
GGACGAAGCUGAUAUCGGA		
GCUAAAGACCAGACGAAGA		
AGGCGAUGGAAGCGGAUUU		
GAAAAGGACAGUAGCUCGA		
ON-TARGETplus Human GNAS siRNA—SMARTpool	Dharmacon	L-010825-00-0005
GCAAGUGGAUCCAGUGCUU		
GCAUGCACCUUCGUCAGUA		
AUGAGGAUCCUGCAUGUUA		
CAACCAAAGUGCAGGACAU		
MDM2 siRNA	Dharmacon	
GACAAAGAAGAGAGUGUGG		[12]
GAAGUUAUUAAAGUCUGUU		[13]
GNAS from short to long isoform primer	Sigma-Aldrich	
GCTGCAAGGAGCAACAGCGATGGTGAGAAGGCAACCAAAG		
CTGCGGGTCCTCTTCGCCGCCCTCTCCATTAAACCCATTAAC		
GNAS from HA to V5 tag primer	Sigma-Aldrich	
CTGCTGGGCCTGGATAGCACCTAAACTCGAGTCTAGAGCGGCC		
CGGGTTCGGAATCGGTTTGCCAGAGCCTCCACCCCCGAG		
GNAS 5X lysine mutation primer	Sigma-Aldrich	
TGAGGCCAACAAAAAGATCGAGAGGCAGCTGCAGAA		
GGTGCTGGAGAATCTGGTAGAAGCACCATTGTGAAG		
GGAGCAACAGCGATGGTGAGAGGGCAACCAAAG		
AGCAAGATCTGCTCGCTGAGAGAGTCCTTGCTG		
GAAAGTCCTTGCTGGGAGATCGAAGATTGAGGACT		
**Recombinant DNA**		
G protein alpha S/GNAS cDNA ORF Clone, Human, C-HA tag	Sino Biological Inc.	HG12069-CY
pcDNA3 Flag beta-2-adrenergic-receptor	Gift from Robert Lefkowitz	[14]
Single Ubiquitin HA tag	Gift from Professor Hemmo Meyer	
**Software and Algorithms**		
ImageJ	Schneider et al., 2012	https://imagej.nih.gov/ij/, accessed on 22 July 2022
GraphPad Prism 8	GraphPad	
EndNote X9	Clarivate Analytics	

## Data Availability

All data are available in the main text or the Supplementary Materials.

## Supplementary Materials

The following supporting information can be downloaded at: https://www.mdpi.com/article/10.3390/cells11162546/s1, Figure S1. MAGED2 prevents internalization of Gαs under hypoxic condition; Figure S2. MAGED2 prevents internalization of Gαs under hypoxic condition in renal cells; Figure S3. MAGED2 prevents internalization of endogenous Gαs under hypoxic condition in cells; Figure S4. MAGED2 promotes cAMP production activity under physical hypoxia; Figure S5. Knockdown of MAGED2 and Gαs; Figure S6. Gαs 5K>R variant is sensitive to cholera toxin induced endocytosis.
