# Supplementary material for: MAGED2 Is Required under Hypoxia for cAMP Signaling by Inhibiting MDM2-Dependent Endocytosis of G-Alpha-S
Source: Cells. 2022 Aug 16;11(16):2546. doi: 10.3390/cells11162546 (PMC9406315; doi:10.3390/cells11162546)
Supplement: Supplementary file 1 [file cells-11-02546-s001.zip › cells-1855217-supplementary.pdf]

Supplementary figures

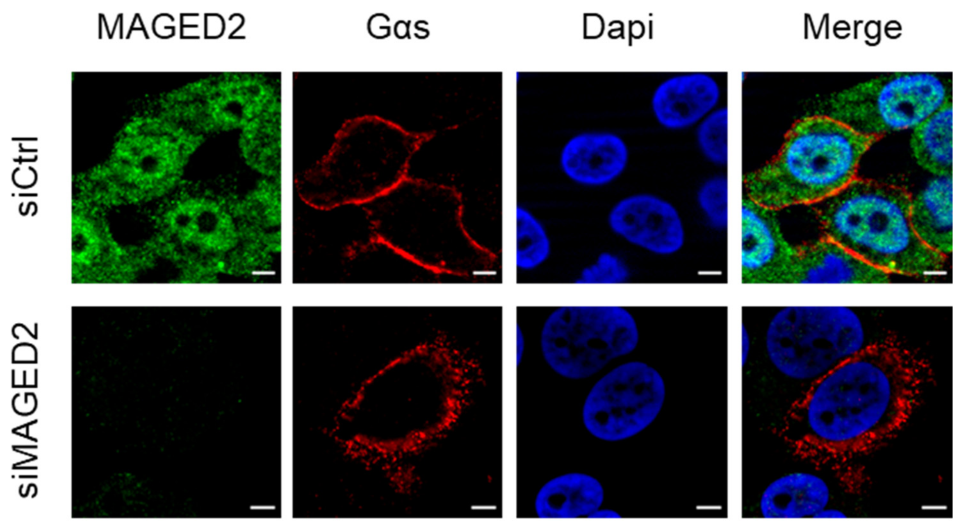

Figure S1. MAGED2 prevents internalization of Gαs under hypoxic condition. Immunolocalization studies of Gαs proteins in presence and absence of MAGED2. HeLa cells were co-transfected with a Gαs-HA construct and control or MAGED2 siRNA. Forty-eight hours post-transfection, growth medium was replaced by DMEM serum free and exposed to physical hypoxia (1% oxygen overnight). Scale bars, 5 μm.

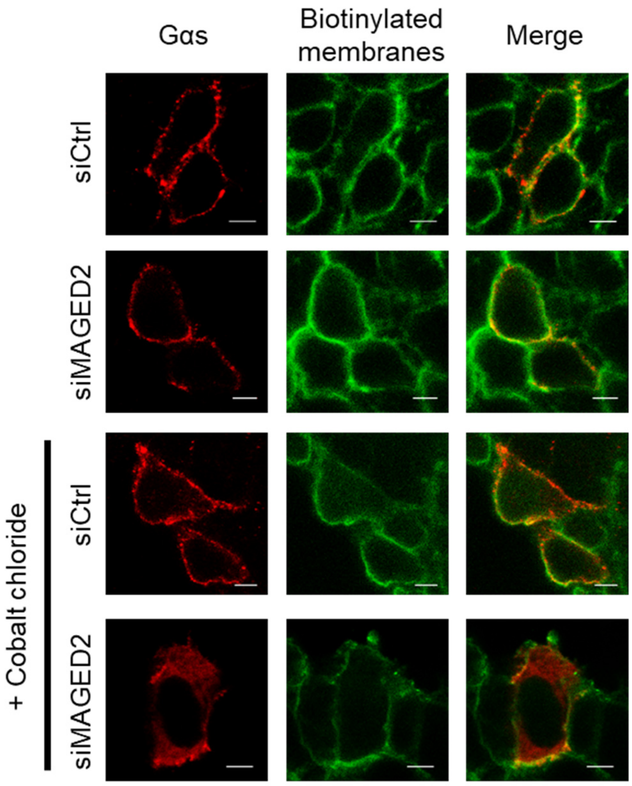

Figure S2. MAGED2 prevents internalization of Gαs under hypoxic condition in renal cells. Immunolocalization studies of Gαs proteins in presence and absence of MAGED2. HEK293 cells were co-transfected with a Gαs-HA construct and control or MAGED2 siRNA. Forty-eight hours post-transfection, growth medium was replaced by DMEM serum free and exposed to chemical hypoxia (300 μM $CoCl_2$), as indicated.

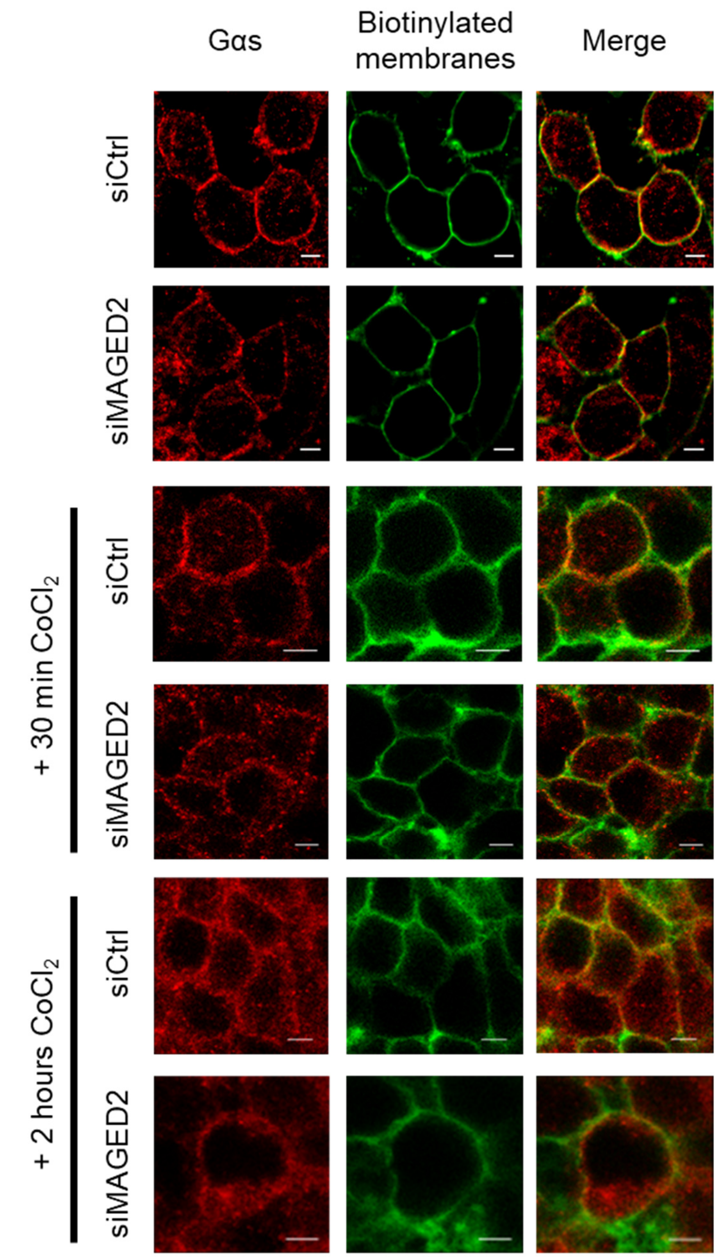

Figure S3. MAGED2 prevents internalization of endogenous Gαs under hypoxic condition in cells. Immunolocalization studies of Gαs proteins in presence and absence of MAGED2. HeLa cells were transfected with a control or MAGED2 siRNA. Forty-eight hours post-transfection, growth medium was replaced by DMEM serum free and exposed to chemical hypoxia (300 μM $CoCl_2$), as indicated.

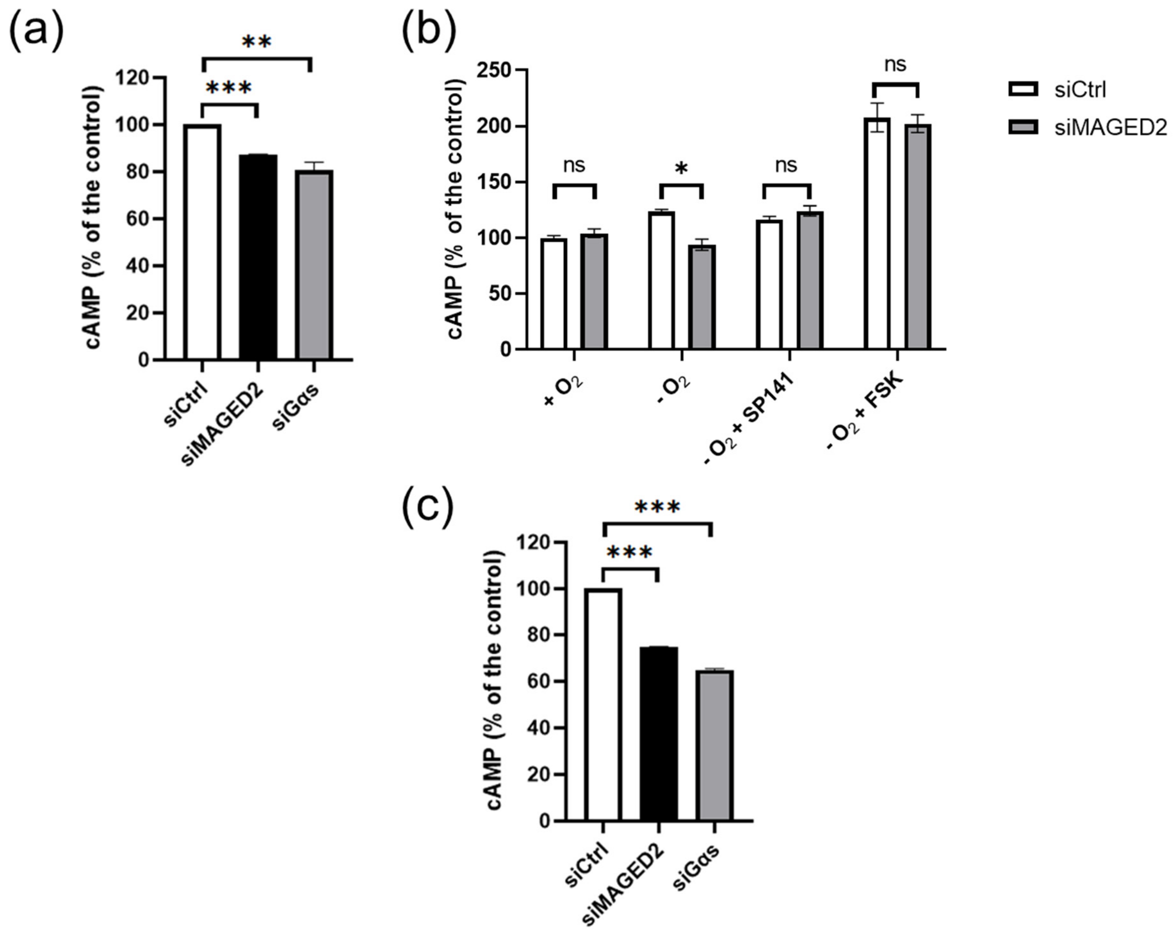

Figure S4. MAGED2 promotes cAMP production activity under physical hypoxia. HeLa (**a** and **b**) and HEK293 (**c**) cells were transfected with control, MAGED2 and Gαs siRNA. Forty-eight hours post-transfection, cells were treated with physical hypoxia overnight in the presence of the phosphodiesterases inhibitor, IBMX 0.5 mM and in the presence or absence of SP141 1 μM or forskolin (FSK) 10 μM, as indicated. Cells were lysed with 0.1 M HCL containing 0.1% Triton X-100 and cAMP was measured by ELISA. Statistical significance was determined by unpaired two tailed Student's t tests (**a** and **c**) or by two-way ANOVA test (**b**). All data are shown as a representative result from three independent experiments (**a** and **c**) or three biological replicates (**b**). Bar graphs show mean ± SEM. * P ≤ 0.05, ** P ≤ 0.01 and *** P ≤ 0.001.

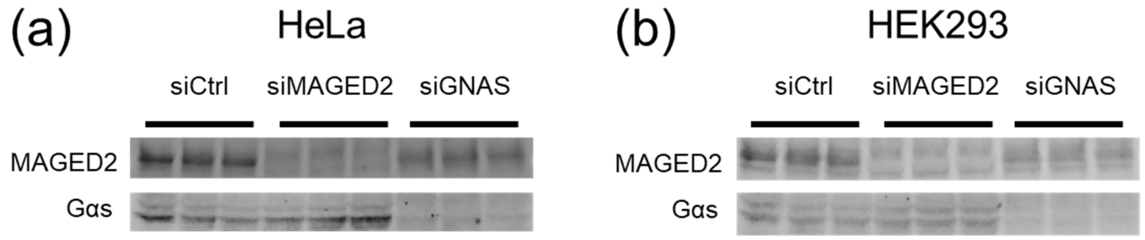

Figure S5. Knockdown of MAGED2 and Gαs markedly reduces expression of MAGED2 and Gαs as revealed by western blotting. These experiments were done in parallel with cAMP and PKA activity measurements (Figure 3 and S4).

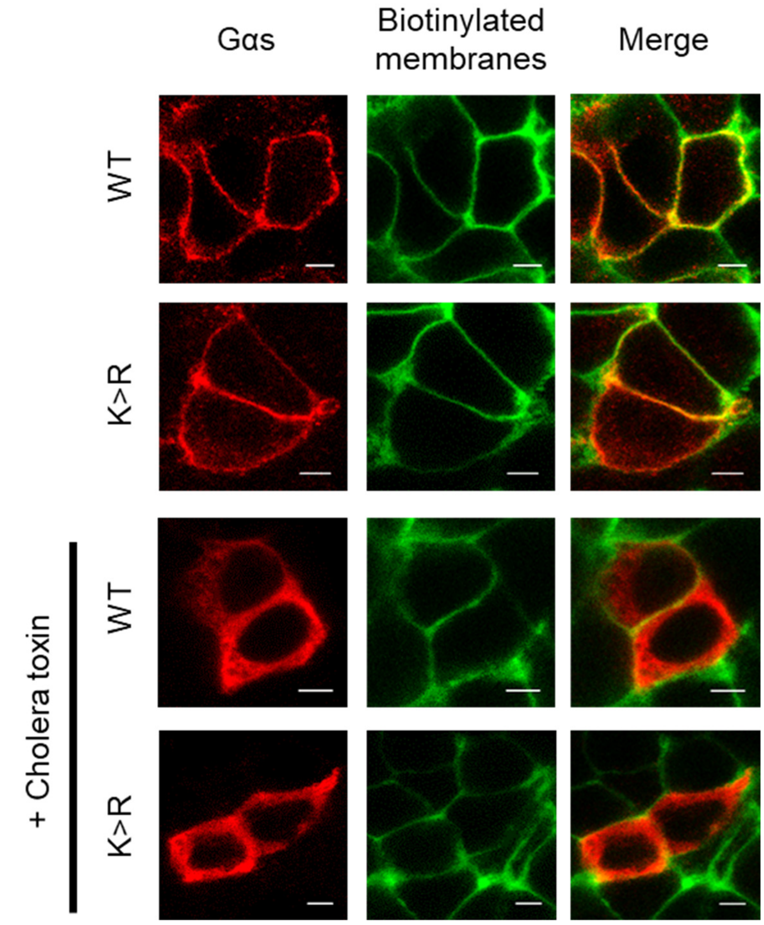

Figure S6. Gαs 5K>R variant is sensitive to cholera toxin induced endocytosis. HeLa cells were transfected with a Gαs-HA WT or 5K>R construct. Forty-eight hours post-transfection, growth medium was replaced by DMEM serum free and exposed to 1 µg/mL of cholera toxin for 4 hours, as indicated. Membrane proteins of HeLa cells were biotinylated at 4 °C. Scale bars, 5 µm.
